# A GABAergic Projection from the Zona Incerta to the Rostral Ventromedial Medulla Modulates Descending Control of Neuropathic Pain

**DOI:** 10.3390/brainsci16010072

**Published:** 2026-01-03

**Authors:** Lijing Zou, Hao Ding, Yujiao Hu, Zhuo Wen, Lina Yu, Min Yan

**Affiliations:** 1 Department of Anesthesiology, Second Affiliated Hospital of Zhejiang University School of Medicine, Hangzhou 310058, China; 12218620@zju.edu.cn (L.Z.); 12518497@zju.edu.cn (Y.H.); 22018239@zju.edu.cn (Z.W.); zryulina@zju.edu.cn (L.Y.); 2 Zhejiang Key Laboratory of Pain Perception and Neuromodulation, Hangzhou 310058, China; 3 Department of Cardiology of the Second Affiliated Hospital, Zhejiang University School of Medicine, Hangzhou 310009, China; 11818166@zju.edu.cn; 4 Cardiovascular Key Laboratory of Zhejiang Province, Hangzhou 310009, China

**Keywords:** neuropathic pain, zona incerta, rostral ventromedial medulla, descending pain modulatory system

## Abstract

**Background**: The rostral ventromedial medulla (RVM) is a central hub of the descending pain modulatory system, yet the inhibitory circuits that regulate its activity during neuropathic pain remain poorly defined. The zona incerta (ZI), a predominantly GABAergic nucleus in the subthalamic region, has been implicated in nociceptive modulation, but its functional connection to the RVM has not been established. **Methods**: A chronic constriction injury (CCI) model was used to induce neuropathic pain. Neuronal activation and circuit connectivity were examined using anatomical tracing and activity mapping. Optogenetic and chemogenetic approaches were employed to selectively manipulate ZI-derived GABAergic projections to the RVM, and mechanical sensitivity was assessed using behavioral assays. **Results**: CCI selectively activated ZI neurons on the ipsilateral side of nerve injury (*p* = 0.0452), which projected to the ipsilateral RVM. Optogenetic activation of ZI-derived terminals in the RVM significantly alleviated CCI-induced mechanical allodynia (*p* = 0.0038), whereas optogenetic inhibition exacerbated pain behaviors (*p* = 0.0183). Consistently, chemogenetic excitation of ZI–RVM neurons attenuated hypersensitivity (*p* < 0.0001), while chemogenetic silencing had the opposite effect (*p* = 0.0015). **Conclusions**: These findings reveal a novel diencephalic-to-brainstem inhibitory pathway that exerts dynamic control over RVM-mediated descending modulation of neuropathic pain.

## 1. Introduction

The rostral ventromedial medulla (RVM) is a central hub of the descending pain modulatory system and plays a pivotal role in the bidirectional control of nociceptive transmission [1,2,3,4,5]. Neurons in the RVM are classically categorized into ON and OFF cells, which respectively facilitate and inhibit pain. Dysregulation of RVM activity has been strongly implicated in the development and maintenance of neuropathic pain [2,4,6]. Although the functional importance of the RVM is well established, how its activity is dynamically shaped by upstream brain circuits under pathological pain conditions remains incompletely understood [7].

Anatomically, the RVM receives convergent inputs from multiple supraspinal regions, including the periaqueductal gray (PAG), hypothalamus, and limbic structures [2,8,9]. While several excitatory projections to the RVM have been described, far less is known about the organization and function of inhibitory afferents that directly regulate RVM output [10,11]. In particular, the identity of GABAergic inputs that exert causal control over RVM-mediated descending modulation during neuropathic pain remains largely unknown. Elucidating the sources and functional roles of these inhibitory pathways is therefore essential for understanding how descending control fails or adapts in chronic pain states.

The zona incerta (ZI) is a predominantly GABAergic nucleus located in the subthalamic region of the diencephalon and has recently been implicated in nociceptive processing [12,13,14]. Accumulating studies indicate that ZI exerts analgesic effects in experimental pain models, yet the downstream circuits through which ZI influences brainstem pain modulatory centers have not been clearly defined [12,15]. Given its anatomical position and inhibitory nature, we hypothesized that ZI may directly regulate RVM activity through a previously uncharacterized descending projection.

In the present study, we demonstrate that ZI provides a direct GABAergic input to the RVM, and we define its functional significance in a model of neuropathic pain. We show that chronic constriction injury (CCI) selectively activates ZI neurons on the ipsilateral side and that these neurons project to the ipsilateral RVM. Using optogenetic and chemogenetic approaches, we further establish that activation of the ZI–RVM pathway attenuates pain hypersensitivity, whereas inhibition of this circuit exacerbates nociceptive behaviors. Together, our findings identify a diencephalic inhibitory input to the RVM and reveal a novel component of the descending pain modulatory system. This ZI–RVM circuit provides a mechanistic link between subthalamic GABAergic activity and brainstem-mediated pain control and may represent a potential therapeutic target for neuropathic pain.

## 2. Materials and Methods

### 2.1. Animals

Wild-type (C57/BL/6J) and transgenic (Vgat-Cre, RRID: IMSR_JAX: 028862) mice were obtained from Shanghai SLAC Laboratory and Jackson Laboratory, respectively. All experimental mice (6–8 weeks) were male to reduce variability introduced by estrous cycle-associated hormonal fluctuations in initial circuit dissection experiments and housed in a standard laboratory environment under a 12/12 h light/dark cycle with free access to food and water. All animal care and experimental procedures were performed in accordance with the National Institutes of Health Guidelines for the Care and Use of Laboratory Animals and were approved by the Experimental Animal Ethics Committee of Zhejiang University (Ethical approval number: ZJU20250404).

### 2.2. CCI Model

A CCI model was established to induce neuropathic pain-like behaviors in mice based on our previously described procedures [16]. Briefly, after being anesthetized with an i.p. injection of sodium pentobarbital (40 mg/kg), the left sciatic nerve trunk of the mouse was exposed by blunt dissection at mid-thigh level, and then four ligatures were tied loosely with 6-0 silk thread around the nerve at 1 mm intervals. The sham-operated animals underwent identical procedures without the ligation of the corresponding nerves.

### 2.3. Viruses and Chemicals

The following were obtained from Wuhan BrainVTA (Wuhan, China): CNO, AAV2/9-hSyn-DIO-NphR-EGFP-WPREs, AAV2/9-hSyn-DIO-ChR2-EGFP-WPREs, AAV2/9-hSyn-DIO-hM3Dq-mCherry-WPREs, AAV2/9-hSyn-DIO-hM4Di-EGFP-WPRE-pA. scAAV2/1-hSyn-Cre-pA, AAV2/9-hSyn-DIO-mCherry-WPRE-pA, and AAV2/9-hSyn-DIO-EGFP-WPRE-pA were obtained from Shanghai Taitool Bioscience (Shanghai, China).

### 2.4. Stereotaxic Surgery

Mice were deeply anesthetized with 5% isoflurane (vol per vol) in oxygen and subsequently maintained with 1% isoflurane during the surgery and virus injection. For cerebral injections, mice were placed in a stereotaxic instrument (RWD, Shenzhen, China) to immobilize the cranium. A dental drill was then used to thin and remove the skull above the target region [17]. The injection coordinates were as follows: 2.15 mm anterior, 1.65 mm lateral, and 4.00 mm ventral for the ZI; 5.80 mm posterior, 0.30 mm lateral, and 5.85 mm ventral for the RVM. A syringe (10 μL, Hamilton, Reno, NV, USA) connected to a glass micropipette (10–15 μm diameter tip) was inserted into the target region to perform the virus injection along with a syringe pump (KD Scientific, 78-8130, Holliston, MA, USA) to control the speed (40 nL/min) and volume. The injection volume was standardized at 150 nL for the cerebral injection. The syringe was left in situ for 10 min after the infusion procedure to allow the viral diffusion. Mice were then housed and allowed to recover and express the virus.

For ZI output tracing, AAV2/9-hSyn-DIO-EGFP-WPRE-pA was unilaterally injected into the ZI of Vgat-Cre mice. For labeling ZI–RVM input neurons, scAAV2/1-hSyn-Cre was injected into the ZI, and AAV2/9-hSyn-DIO-mCherry-WPRE-pA was injected into the RVM of wild-type mice [18]. Four weeks (one week for the CTB 555) later, mice were euthanized, and ZI and RVM sections were collected for imaging.

For axon terminal photoactivation or photoinhibition, AAV2/9-hSyn-DIO-NphR-EGFP-WPREs or AAV2/9-hSyn-DIO-ChR2-EGFP-WPREs (with AAV-DIO-EGFP as a control) were unilaterally injected into the ZI of wild-type mice. Three weeks later, optical fibers were placed 150 μm above the RVM injection site. Mice were allowed one week to recover before behavioral testing.

To manipulate ZI–RVM input neurons, scAAV2/1-hSyn-Cre was injected into the ZI, and AAV2/9-hSyn-DIO-hM3Dq-mCherry-WPREs or AAV2/9-hSyn-DIO-hM4Di-EGFP-WPRE-pA into the RVM. Behavioral tests were performed 40 min after CNO (4 mg/kg) or saline injection, 4 weeks post-virus injection.

### 2.5. Immunohistochemistry and Imaging

Mice were sacrificed and transcardially perfused with 4% paraformaldehyde (PFA) in phosphate-buffered saline (PBS). Brains were removed and post-fixed overnight in 4% PFA and then immersed in 30% sucrose at 4 °C for 48 h. Coronal sections were cut at 40 μm containing target brain regions on a cryostat (Leica CM1900). Brain sections were washed with PBS three times and blocked with 10% goat serum dissolved in 0.3% Triton-X 100 in PBS for 1 h at room temperature followed by incubation with primary antibodies (Fos: 1:10,000, rabbit, Synaptic Systems, Cat# 226008, Goettingen, Germany) overnight at 4 °C. Sections were then rinsed three times with PBS and incubated with corresponding secondary antibodies (1:2000, Earthox, Cat# E031210, San Francisco, CA, USA) at room temperature for 2 h, and the nuclei were counterstained with DAPI (VECTASHIELD, Newark, CA, USA) [17]. Sections were mounted onto glass slides and imaged with an Olympus VS120 microscope (Tokyo, Japan) or a Nikon A1 confocal microscope (Tokyo, Japan) as required.

### 2.6. In Vivo Optogenetic Manipulation

These procedures were performed as previously described with minor modifications [17]. The optical fiber was connected to an intelligent laser system (Inper, Aurora-200, Hangzhou, China) to perform the light control. As for the activation experiment, a 473 nm wavelength (blue) at 5 mW with a 10 ms pulse width was used, while a 589 nm wavelength (yellow) at 5 mW with a continuous mode was employed for the inhibition study.

### 2.7. Behavioral Tests

All mice subjected to behavioral tests were handled extensively and habituated to the test environments for at least 3 days to minimize stress. Additionally, the room temperature and humidity were kept stable in all experiments. Behavioral tests were performed by experimenters blinded to viral condition and/or stimulation/drug treatment.

#### 2.7.1. Von Frey Test

The mechanical hypersensitivity of mice was evaluated by the PWT, which is defined as a response to von Frey filament stimulation. Before the test, each animal was introduced to an individual Plexiglas chamber on an elevated mesh floor and allowed to acclimate for 1.5 h. The hind paw of the mouse was perpendicularly stimulated with a series of calibrated von Frey filaments starting from 0.16 g. A positive pain response was defined as a withdrawal or flinching of the hind paw after the von Frey filament stimulation. The next greater von Frey filament was applied when a negative response was observed, while the next weaker von Frey filament was chosen if a positive response occurred. Each mouse underwent von Frey filament testing to determine the 50% PWT based on Dixon’s up-down method [19]. Baseline mechanical thresholds (von Frey) were measured after 3 weeks of stereotactic surgery. For neuropathic pain experiments, von Frey testing was performed after 7 days of CCI surgery.

#### 2.7.2. Open Field Test

The locomotor activity of mice was evaluated by the open field test. Mice were placed in the center of a polystyrene chamber that was 40 cm long, 40 cm wide, and 40 cm high to explore freely for 5 min. The track of each mouse was videotaped and analyzed by the ANY-maze. Experiments were conducted under dimly illuminated conditions, with the open field cleaned with 75% ethanol between each test. The center referred to the central 20 × 20 cm area. The total distance traveled was used to measure locomotion, and the percentage of time spent in the central area was used as an evaluation of anxiety-like behavior.

#### 2.7.3. Elevated Plus Maze

The elevated plus maze apparatus was 50 cm above the floor and consisted of two opposing open arms (30 × 5 cm), two opposing closed arms (30 × 5 × 16 cm), and a central area (5 × 5 cm). Mice were placed individually in the center of the apparatus facing a closed arm and allowed to explore freely for 5 min. Movement and the percentage of time in the open arms were recorded and analyzed with the ANY-maze software (Stoelting Co., Wood Dale, IL, USA).

### 2.8. Quantification and Statistical Analysis

Statistical analysis was conducted blindly with respect to experimental conditions. Data were presented as mean ± SEM and analyzed by GraphPad Prism software (version 6.0). Comparisons between two groups were determined by a two-tailed unpaired *t*-test. Repeated-measures two-way ANOVA followed by the Sidak post hoc test was used for multiple group data comparisons. Statistical significance was indicated as * *p* < 0.05, ** *p* < 0.01, *** *p* < 0.001 and **** *p* < 0.0001.

## 3. Results

### 3.1. Ipsilateral ZI Is Activated by Nerve Injury and Innervates the RVM

**Figure 1:**
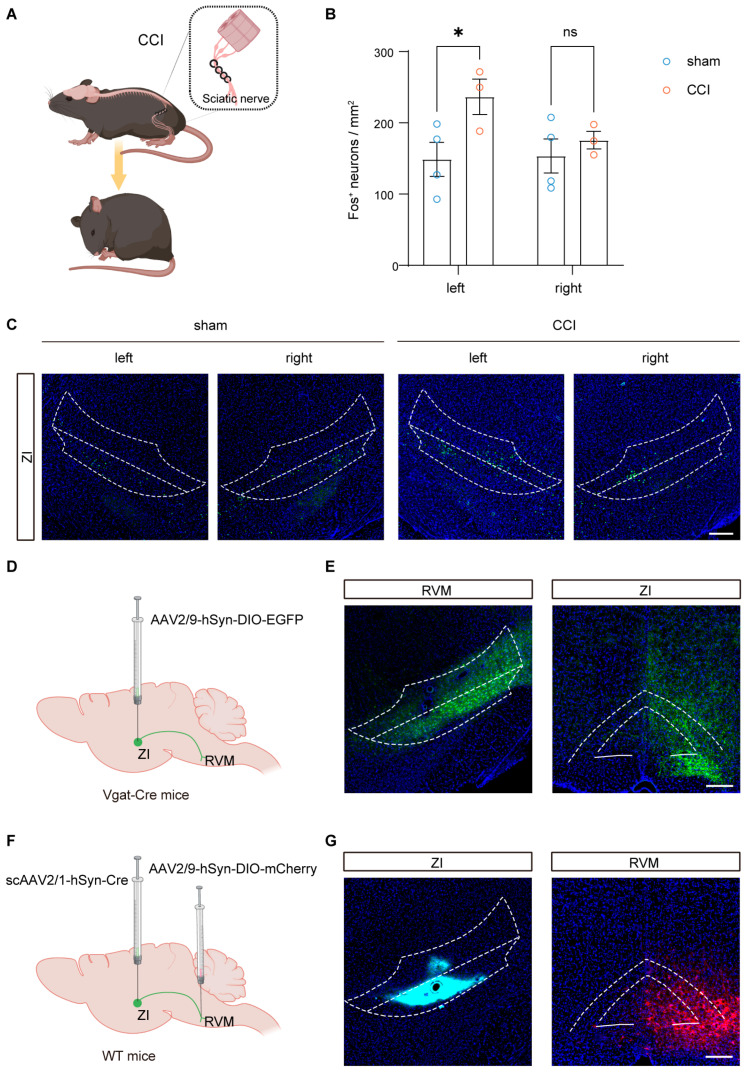
ZI is activated in neuropathic pain and projects to RVM. (**A**) Schematic of the CCI model. (**B**) Quantitative analysis of Fos expression in the bilateral ZI of sham and CCI mice (*n* = 4 mice for the sham group and 5 mice for the CCI group). (**C**) Representative images of Fos expression in the bilateral ZI of sham and CCI mice. Scale bar, 200 μm. (**D**) Schematic of strategy expressing DIO-EGFP in ZI^GABA^ neurons of Vgat-Cre mice to observe projections into the RVM. (**E**) Representative image of the injection site in the ZI (**left**) and EGFP-labeled fibers in the RVM (**right**). Scale bar, 200 μm. (**F**) Schematic of the monosynaptic anterograde tracing strategy from the ZI to the RVM of WT mice. (**G**) Representative images showing scAAV2/1-hSyn-Cre (mixed with CTB 647) expression in the ZI (**left**) and DIO-mCherry in the RVM (**right**). Scale bar, 200 μm. ns *p* > 0.05, * *p* < 0.05. Repeated-measures two-way ANOVA followed by Sidak post hoc test. Data were presented as mean ± SEM. White linear demarcations define the extent of the associated brain areas.

To examine whether the ZI participates in neuropathic pain processing, we first assessed neuronal activation in the ZI following chronic constriction injury (CCI) (Figure 1A). Quantification of Fos expression revealed a significant increase in Fos-positive neurons in the ipsilateral ZI of CCI mice compared with sham controls, whereas no significant change was observed in the contralateral ZI (Figure 1B,C), indicating lateralized activation of the ZI following nerve injury. We next investigated whether ZI GABAergic (ZI^GABA^) neurons project to the RVM. To this end, DIO-EGFP was selectively expressed in ZI^GABA^ neurons in Vgat-Cre mice (Figure 1D). EGFP-labeled axons were observed in the RVM, confirming a direct anatomical projection from ZI^GABA^ neurons to the RVM (Figure 1E). To further validate this connection, we employed a monosynaptic anterograde tracing strategy based on scAAV2/1-mediated Cre delivery combined with DIO-mCherry expression in the RVM (Figure 1F). Robust mCherry expression was detected in the RVM following Cre delivery in the ZI (Figure 1G), providing additional evidence for a direct ZI–RVM projection.

### 3.2. Optogenetic Inhibition of the ZI–RVM Pathway Induces Mechanical Allodynia

**Figure 2:**
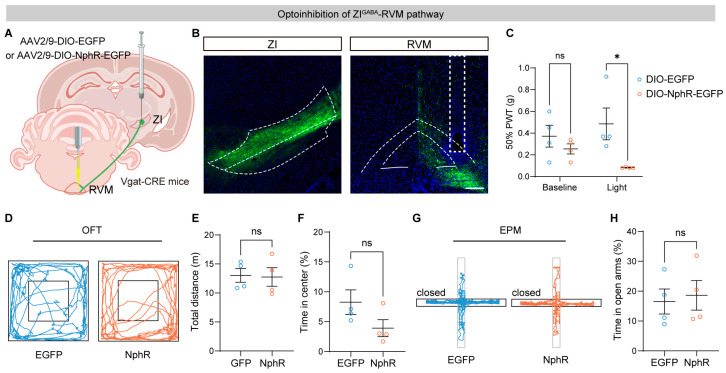
Optoinhibition of the ZI–RVM circuit induces mechanical allodynia. (**A**) Schematic of the strategy for optogenetic inhibition of RVM-projecting ZI^GABA^ terminals. (**B**) Representative images of DIO-NphR-EGFP expression in the ZI^GABA^ (**left**) and RVM with the fiber track above (**right**). Scale bar, 200 μm. White linear demarcations define the extent of the associated brain areas, while the rectangular white delineations designate the site of the insert core. (**C**) Effects of optogenetic inhibition of the ZI^GABA^-RVM circuit on mechanical thresholds of ipsilateral hind paws (*n* = 4 mice per group). (**D**–**H**) Effects of optoinhibition of the ZI^GABA^-RVM pathway on locomotion activity and anxiety level: example tracts (**D**), total distance (**E**), and time spent in center (**F**) in the open field test (OFT), and example tracts (**G**) and time in open arms (**H**) in the elevated plus maze (EPM) test. ns *p* > 0.05, * *p* < 0.05. Repeated-measures two-way ANOVA followed by Sidak post hoc test for (**C**) and unpaired *t*-test for (**E**,**F**,**H**). Data were presented as mean ± SEM.

To determine the functional role of the ZI–RVM projection in nociceptive processing, we optogenetically inhibited RVM-projecting ZI^GABA^ terminals using DIO-NpHR-EGFP in Vgat-Cre mice (Figure 2A). Efficient viral expression in ZI^GABA^ neurons and optical fiber placement above the RVM were confirmed histologically (Figure 2B). Optogenetic inhibition of the ZI^GABA^-RVM pathway significantly reduced the mechanical withdrawal threshold of the ipsilateral hind paw, indicating the development of mechanical allodynia (Figure 2C). To assess whether behavioral changes were secondary to alterations in emotional or locomotor states, mice were subjected to the open field test (OFT) and elevated plus maze (EPM). No significant differences were observed in total distance traveled, time spent in the center of the open field, or time spent in open arms between experimental and control groups (Figure 2D–H), suggesting that inhibition of the ZI–RVM pathway selectively affects nociceptive sensitivity without altering anxiety-like behavior or locomotor activity.

### 3.3. Chemogenetic Inhibition of ZI–RVM Neurons Promotes Pain Hypersensitivity

**Figure 3:**
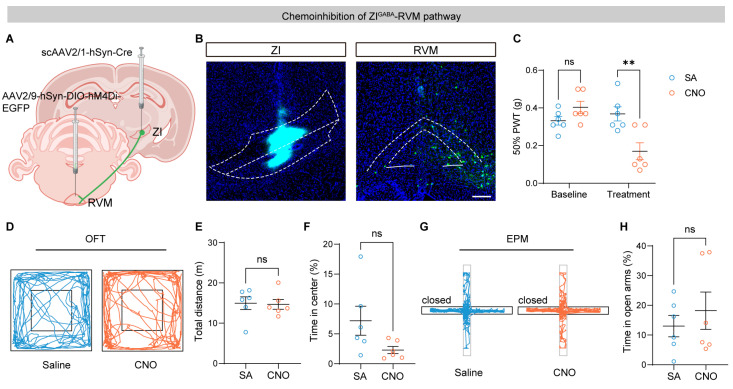
Chemoinhibition of the ZI–RVM circuit induces mechanical allodynia. (**A**) Schematic of the strategy for chemogenetic inhibition of ZI–RVM neurons. (**B**) Representative images of scAAV2/1-hSyn-Cre (mixed with CTB 647) expression in the ZI (**left**) and DIO-hM4Di-EGFP in the RVM (**right**). Scale bar, 200 μm. White linear demarcations define the extent of the associated brain areas. (**C**) Effects of chemogenetic inhibition of the ZI–RVM circuit on mechanical thresholds of ipsilateral hind paws (*n* = 6 mice per group). (**D**–**H**) Effects of chemoinhibition of the ZI–RVM pathway on locomotor activity and anxiety level: example tracts (**D**), total distance (**E**), and time spent in center (**F**) in the open field test (OFT), and example tracts (**G**) and time in open arms (**H**) in the elevated plus maze (EPM) test. ns *p* > 0.05, ** *p* < 0.01. Repeated-measures two-way ANOVA followed by Sidak post hoc test for (**C**) and unpaired *t*-test for (**E**,**F**,**H**). Data were presented as mean ± SEM.

To independently validate the effects of suppressing the ZI–RVM pathway, we chemogenetically inhibited RVM-projecting ZI neurons by expressing hM4Di in the RVM following Cre delivery from the ZI (Figure 3A,B). Administration of CNO significantly decreased mechanical thresholds in the ipsilateral hind paw compared with saline controls, demonstrating increased pain sensitivity (Figure 3C). Consistent with the optogenetic results, inhibition of ZI–RVM neurons did not alter locomotor activity or anxiety-related behavior as assessed by the OFT and EPM (Figure 3D–H). These findings confirm that suppression of the ZI–RVM pathway is sufficient to induce mechanical allodynia without confounding effects on affective or motor behavior.

### 3.4. Optogenetic Activation of the ZI–RVM Pathway Alleviates Neuropathic Pain

**Figure 4:**
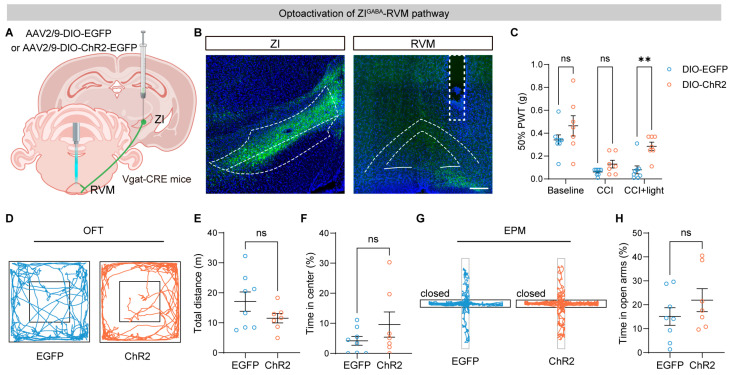
Optoactivation of the ZI–RVM circuit alleviates pain hypersensitivity of CCI mice. (**A**) Schematic of the strategy for optogenetic activation of RVM-projecting ZI^GABA^ terminals. (**B**) Representative images of DIO-ChR2-EGFP expression in the ZI^GABA^ (**left**) and RVM with the fiber track above (**right**). Scale bar, 200 μm. White linear demarcations define the extent of the associated brain areas, while the rectangular white delineations designate the site of the insert core. (**C**) Effects of optogenetic activation of the ZI^GABA^-RVM circuit on mechanical thresholds of injured hind paws (*n* = 8 mice for the EGFP group and *n* = 7 mice for the ChR2 group). (**D**–**H**) Effects of optoactivation of the ZI^GABA^-RVM pathway on locomotion activity and anxiety level: example tracts (**D**), total distance (**E**), and time spent in the center (**F**) in the open field test (OFT), and example tracts (**G**) and time in open arms (**H**) in the elevated plus maze (EPM) test. ns *p* > 0.05, ** *p* < 0.01. Repeated-measures two-way ANOVA followed by Sidak post hoc test for (**C**) and unpaired *t*-test for (**E**,**F**,**H**). Data were presented as mean ± SEM.

We next examined whether activation of the ZI–RVM circuit could attenuate pain hypersensitivity in CCI mice. Channelrhodopsin-2 (ChR2) was selectively expressed in ZI^GABA^ neurons, and an optical fiber was implanted into the RVM (Figure 4A,B). After 3 weeks, the mice were tested with von Frey fibers to establish the baseline mechanical sensitivity and then received CCI surgery. Behavioral tests were performed after 7 days of CCI surgery (Supplementary Figure S1C). Optogenetic activation of the ZI^GABA^-RVM pathway significantly increased the mechanical threshold of the injured hind paw, indicating reduced pain sensitivity (Figure 4C). Behavioral analyses using OFT and EPM revealed no significant differences in locomotion or anxiety levels between stimulation and control conditions (Figure 4D–H), excluding nonspecific behavioral effects. These data indicate that selective activation of the ZI–RVM pathway produces a robust analgesic effect under neuropathic pain conditions.

### 3.5. Chemogenetic Activation of ZI–RVM Neurons Attenuates Pain Hypersensitivity

**Figure 5:**
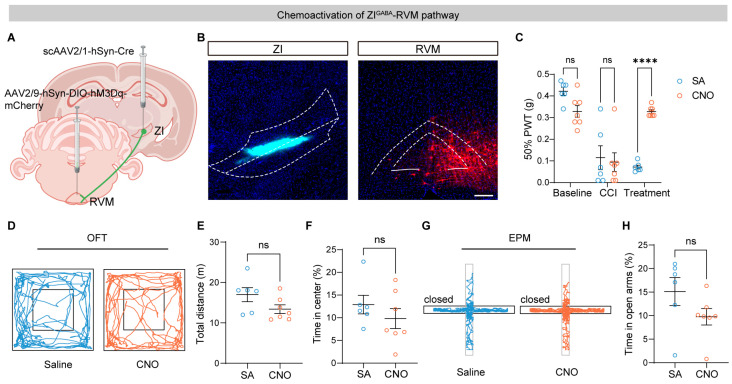
Chemoactivation of the ZI–RVM circuit alleviates pain hypersensitivity of CCI mice. (**A**) Schematic of the strategy for chemogenetic activation of ZI–RVM neurons. (**B**) Representative images of scAAV2/1-hSyn-Cre (mixed with CTB 647) expression in the ZI (**left**) and DIO-hM3Dq-mCherry in the RVM (**right**). Scale bar, 200 μm. White linear demarcations define the extent of the associated brain areas. (**C**) Effects of chemogenetic activation of the ZI–RVM circuit on mechanical thresholds of injured hind paws (*n* = 6 mice for the SA group and *n* = 7 mice for the CNO group). (**D**–**H**) Effects of chemoactivation of the ZI–RVM pathway on locomotion activity and anxiety level: Example tracts (**D**), total distance (**E**), and time spent in the center (**F**) in the open field test (OFT), and example tracts (**G**) and time in open arms (**H**) in the elevated plus maze (EPM) test. ns *p* > 0.05, **** *p* < 0.0001. Repeated-measures two-way ANOVA followed by Sidak post hoc test for (**C**) and unpaired *t*-test for (**E**,**F**,**H**). Data were presented as mean ± SEM.

To further confirm the analgesic role of the ZI–RVM pathway, we activated RVM-projecting ZI neurons using hM3Dq-mediated chemogenetic stimulation (Figure 5A,B, Supplementary Figure S1D). Compared with saline treatment, CNO administration significantly elevated mechanical thresholds in CCI mice (Figure 5C), consistent with reduced pain sensitivity. Similarly to optogenetic activation, chemogenetic excitation of the ZI–RVM pathway did not significantly affect locomotor activity or anxiety-related behavior (Figure 5D–H). Together, these results demonstrate that activation of ZI–RVM neurons is sufficient to relieve neuropathic pain without producing detectable side effects on general behavior.

## 4. Discussion

In this study, we identify a previously uncharacterized GABAergic projection from the zona incerta (ZI) to the rostral ventromedial medulla (RVM) and demonstrate that this pathway plays a critical role in descending modulation of neuropathic pain. Although the RVM is a central component of the descending pain modulatory system, most prior work has emphasized excitatory inputs from structures such as the periaqueductal gray (PAG) and limbic regions, whereas inhibitory afferents remain poorly understood [2,6,20,21,22]. Our findings extend this framework by establishing the ZI as a diencephalic source of inhibitory control over RVM-mediated pain regulation.

Previous studies have implicated the ZI in sensory gating and analgesia, showing that activation of ZI GABAergic neurons suppresses nocifensive behaviors and reduces thalamocortical sensory transmission [23,24,25,26]. However, the downstream targets through which the ZI mediates these effects have not been clearly defined. Here, using a combination of anterograde tracing and projection-specific manipulations, we show that ZI GABAergic neurons project to the RVM and that this projection is both necessary and sufficient for controlling mechanical hypersensitivity. Optogenetic and chemogenetic activation of the ZI–RVM pathway alleviated pain behaviors, whereas inhibition reliably induced mechanical allodynia. These results demonstrate that ZI–RVM neurons constitute a key inhibitory component of the descending modulatory network. Furthermore, the ZI likely engages multiple descending modulatory routes, including PAG-related disinhibitory mechanisms. While our projection-specific manipulations establish a causal and sufficient role for the direct ZI–RVM pathway, future studies dissecting ZI–PAG interactions will be required to determine how these parallel circuits integrate to shape net descending analgesia.

A notable feature of this circuit is its strong lateralization. We observed robust Fos activation selectively in the ipsilateral ZI following chronic constriction injury (CCI), and tracing experiments confirmed that ZI neurons preferentially innervate the ipsilateral RVM. This contrasts with classical descending pathways such as the PAG-RVM projection, which are largely bilateral [27,28]. The ipsilateral organization of the ZI–RVM circuit suggests that the ZI provides spatially precise inhibitory control that matches the side-specific nature of peripheral nerve injury [29]. Such lateralized descending inhibition may allow the ZI to selectively counterbalance hypersensitivity on the injured side without broadly suppressing bilateral sensory processing. This spatial fidelity aligns with emerging evidence highlighting functional modularity and anatomical specificity within ZI subregions.

Our findings also complement the classical model of descending pain modulation. While PAG-driven inhibition is well established, accumulating evidence indicates that diencephalic structures, including the hypothalamus and ZI, play broader roles in shaping brainstem output [30,31]. By identifying a direct inhibitory route from the ZI to the RVM, our study introduces an additional layer of control within the descending system and suggests that ZI activity may interact with specific RVM neuronal subtypes, such as ON and OFF cells. Future electrophysiological studies will be valuable for determining whether ZI-derived GABAergic inputs preferentially facilitate OFF-cell activity, inhibit ON cells, or modulate local GABAergic microcircuits within the RVM.

The translational implications of this circuit are noteworthy. Deep brain stimulation (DBS) of the posterior subthalamic region, covering the ZI, has shown analgesic benefits in certain conditions, although its mechanisms remain unclear [32,33]. Our results provide a mechanistic rationale for these observations, suggesting that enhancing ZI-derived inhibitory input to the RVM may represent a targeted strategy for restoring descending inhibition in neuropathic pain. Identifying upstream regulators of ZI GABAergic neurons may further broaden therapeutic potential. Given the established preclinical and clinical observation that ZI stimulation selectively reduces human heat pain, a notable limitation of our work is whether this pathway modulates other pain dimensions, which requires further investigation [34]. Our study exclusively focused on mechanical pain, so this limitation highlights the imperative for future studies to systematically investigate its effects across sensory modalities (e.g., heat pain and acetone test) and affective aspects (e.g., conditioned place preference).

In addition, several limitations should be acknowledged. First, our study did not have a formal a priori power analysis; the sample size was based on prior studies [16,35]. Second, we did not characterize the specific RVM cell types targeted by ZI inputs and validate the synaptic mechanisms and postsynaptic cell-type specificity in the RVM using electrophysiological methods. Third, we observed lateralized innervation of this pathway but did not deeply explore it. We acknowledge that contralateral effects could exist, and future experiments are required to examine bilateral behavioral outcomes and bilateral circuit dynamics. Additionally, validating this circuit in other species and in female mice will be essential for assessing translational relevance.

## 5. Conclusions

In summary, we reveal a spatially precise GABAergic diencephalic-to-brainstem circuit that exerts powerful control over neuropathic pain. These findings enrich the current model of descending pain modulation and highlight the ZI–RVM pathway as a promising target for therapeutic intervention.

## Data Availability

The original data presented in the study are openly available in Figshare at 10.6084/m9.figshare.30774881.

## Supplementary Materials

The following supporting information can be downloaded at: https://www.mdpi.com/article/10.3390/brainsci16010072/s1, Figure S1: The experimental time points of Figure 2, Figure 3, Figure 4 and Figure 5.

## Abbreviations

The following abbreviations are used in this manuscript:

RVM	Rostral ventromedial medulla
ZI	Zona incerta
CCI	Chronic constriction injury
PAG	Periaqueductal gray
OFT	Open field test
EPM	Elevated plus maze
PWT	Paw withdrawal threshold
