# Supplementary figures and images for: A GABAergic Projection from the Zona Incerta to the Rostral Ventromedial Medulla Modulates Descending Control of Neuropathic Pain
Source: Brain Sci. 2026 Jan 3;16(1):72. doi: 10.3390/brainsci16010072 (PMC12838893; doi:10.3390/brainsci16010072)

**A**

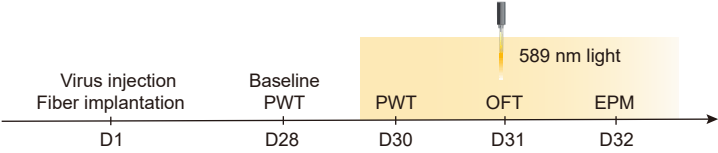

**B**

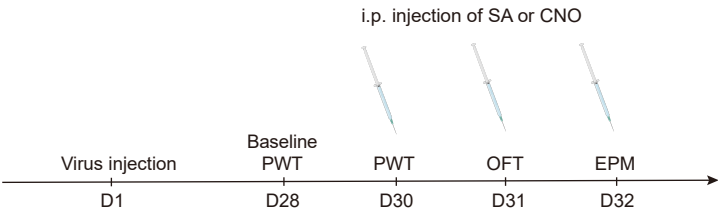

**C**

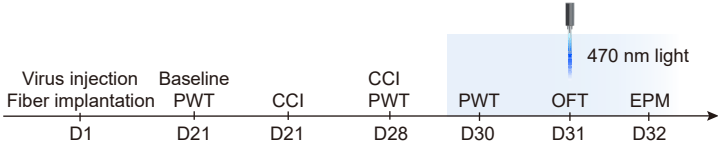

**D**

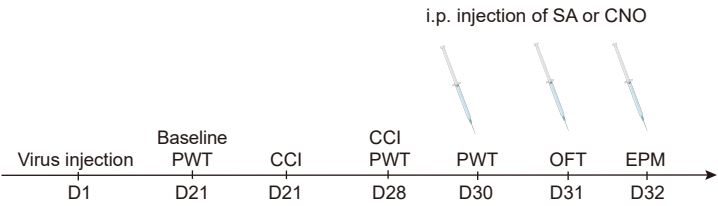

Supplement: Supplementary file 1 [file brainsci-16-00072-s001.zip › brainsci-4056852-supplementary.pdf]
